# Diagnostic Value of Plasma miR-145 and miR-185 as Targeting of the APRIL Oncogene in the B-cell Chronic Lymphocytic Leukemia

**DOI:** 10.31557/APJCP.2021.22.1.111

**Published:** 2021-01

**Authors:** Malihe Bagheri, Behzad Khansarinejad, Ghasem Mosayebi, Alireza Moradabadi, Mahdieh Mondanizadeh

**Affiliations:** 1 *Department of Biotechnology and Molecular Medicine, Arak University of Medical Sciences, Arak, Iran. *; 2 *Department of Microbiology and Immunology, Arak University of Medical Sciences, Arak, Iran. *; 3 *Department of Hematology and Blood Banking, Arak University of Medical Sciences, Arak, Iran.*; 4 *Molecular and Medicine Research Center, Arak University of Medical Sciences, Arak, Iran. *

**Keywords:** B-CLL, miRNAs, biomarker, APRIL

## Abstract

**Background:**

Chronic lymphocytic leukemia (CLL) is one of the most common hematologic malignancy in adults worldwide. This cancer has a poor prognosis at different stages. So, the identification of new biomarkers is important for diagnosis of B-CLL. Considering the oncogenic role of APRIL molecule in this leukemia as well as the regulatory role of miRNAs in different signaling pathways, the present study evaluated the miRNAs targeting APRIL gene in B-CLL.

**Methods:**

The miRNAs were predicted and selected using bioinformatics algorithms. A total of 80 plasma samples were subjected to RNA extraction and synthesis of cDNA. The expressions levels of predicted miRNAs and APRIL gene in plasma of B-CLL patients and healthy individuals were assessed by Real time PCR analysis. ROC analysis was performed to investigate the role predicted miRNAs as novel biomarkers in diagnosis of B-CLL.

**Results:**

The results of the prediction showed that miR-145-5p and miR-185-5p target the APRIL gene. The expression level of APRIL gene was strikingly higher in plasma of B-CLL patients than in the healthy individuals (102, P= 0.001). On the other hand, expression levels of miR-145-5p and miR-185-5p were strikingly lower in B-CLL patients than in the healthy individuals (0.07, P= 0.001) (0.29, P= 0.001). Also, ROC curve analyses demonstrated that miR-145-5p and miR-185-5p are specific and sensitive and may serve as new biomarkers for the detection of B-CLL. (AUC; 0.95, sensitivity; %90) (AUC; 0.87, sensitivity; %63).

**Conclusion:**

These data suggest that miR-145-5p and miR-185-5p target the APRIL gene and might have a role in diagnosis of B-CLL. Therefore, these two miRNAs can be served as a novel and potential biomarker for detection of B-CLL.

## Introduction

Chronic lymphocytic leukemia (CLL) is one of the most common hematologic malignancy in adults worldwide, that makes up approximately 30% of all blood cancers (Rodrigues et al., 2016). In this leukemia, mature and monoclonal B lymphocyte cells with specific surface markers such as CD 5, 19 and 23 accumulate in the peripheral blood (PB), lymphoid organs, bone marrow (BM) and spleen (Lopez-Guerra and Colomer, 2010). CLL is often diagnosed using a variety of methods such as complete blood count (CBC), differential white blood cell count, morphological assessment of blood smear and immunophenotyping of PB (Hallek et al., 2008; Eichhorst et al., 2015; Strati and Shanafelt, 2015). Besides of these diagnostic methods, it is difficult to diagnose CLL due to the lack of specific sings (Mirzaei et al., 2018a). So, the identification of suitable biomarkers for better diagnosis and appropriate treatment is important. 

At present, microRNAs (miRNAs) with properties such as tissue specificity, rapid release rate and plasma stability have been identified as non-invasive biomarkers in diagnosis and treatment of CLL (Mitchell et al., 2008; Moussay et al., 2011; Wang et al., 2017). These molecules regulate gene expression through target mRNA degradation or inhibition in translation level (Mirzaei et al., 2018b). They Also play a critical role in various processes of physiological and pathological such as cell growth and differentiation, metabolism, apoptosis, angiogenesis and tumorigenesis (Simonian et al., 2018). The structure and function of miRNAs suggest that the expression of many miRNAs in cancerous tissues changes abnormally compared to normal tissue (Lu et al., 2005). Therefore, altering the expression profile of miRNAs can be used for the detection of a wide range of diseases (Ardekani and Naeini, 2010). Further, miRNAs are associated with signaling pathways that frequently change in different cancers such as CLL (Rushworth et al., 2012). 

Among signaling pathways involved in CLL pathogenesis, NF-kB signaling pathway has an essential role in disease development (Ferrer and Montserrat, 2018). In alternative (non-canonical) NF-kB signaling pathway, APRIL (a proliferation-inducing ligand) as one of the members of the tumor necrosis factor (TNF) family bind to tumor necrosis factor receptor (TNFR) such as TACI (transmembrane activator and calcium modulator and cyclophilin ligand interactor) and BCMA (B-cell maturation antigen) and causes proliferation, survival and protection CLL cells from apoptosis (Mackay and Ambrose, 2003; Haiat et al., 2006). APRIL is a soluble molecule secreted from the Golgi apparatus (Lopez-Fraga et al., 2001). This molecule not detectable in normal tissues but is overexpressed in tumor tissues. The various studies have shown that the level of APRIL in the serum of CLL patients are significantly higher than in normal individuals (Hahne et al., 1998a; Tecchio et al., 2011). 

Subsequently, considering the role of APRIL molecule in the pathogenesis of CLL and also the regulatory role of miRNAs in many signaling pathways, the aim of present study was to identify miRNAs targeting APRIL mRNAs in B-CLL.

## Materials and Methods

*Patients and Clinical samples*

In total, 80 plasma samples were collected from Valiasr Hospital (Arak, Iran) and stored at -70˚C before analysis. The collected samples included 40 samples of healthy individuals as control group and 40 samples of patients. All patients diagnosed as B-CLL by FAB criteria with an expert hemato oncologist. In diagnosed of B-CLL patients, European Group for the Immunological Characterization of Leukemias (EGIL) suggested the flow cytometry marker to diagnosed positive for CD5, CD19, CD20 and CD23, the level of surface CD20, and CD79 are low in compare of normal B-cell also, B-cells are negative for CD13, CD117 and other myeloid linage markers. Also, the B-CLL group did not receive any conventional treatment (surgery, chemotherapy and radiotherapy). In both groups, samples were taken from 20 males and 20 females, with a median age of 68 years (range, 60 to 76 years) in the group of patients and 70.5 years (range, 62-79 years) in the group of healthy individual. All patients and healthy volunteers provided written informed consent and the study was approved by the Ethics Committee of Arak University of Medical Sciences, in accordance with the declaration of Helsinki. The characteristics of the patients under study and the control group are shown in Table 1. The study approved in Arak University of medical science ethical committee and The Ethic Approval Code is IR.ARAKMU.REC.1395.418.

*Prediction of miRNAs targeting APRIL gene*

Prediction of miRNAs targeting APRIL gene was conducted by bioinformatics algorithms according to rules of complementary base pairs between miRNA and mRNA. Therefore, miRNA binds to the 3’ untranslated region (3’-UTR) in the target mRNA. In this way, the sequences of APRIL gene with official name of TNFSF13 (TNF superfamily member 13) were obtained using the NCBI (http://www.ncbi.nlm.nih.gov/gene). Then, the miRNAs targeting APRIL gene were predicted by various bioinformatics tools such as miRanda (http://www.microrna.org/microrna/home.do), TargetScan (http://www.TargetScan), mirWalk (http://www.umm.uni-heidelberg.de/apps/zmf/mirwalk), DIANA (http://diana.imis.athena-innovation.gr/DianaTools/index.php?r=microT_CDS/index), miRTarBase (mirtarbase.mbc.nctu.edu.tw/php/index.php), miRDB (http://mirdb.org/), miRdSNP (http://mirdsnp.ccr.buffalo.edu/search.php), miRecords (http://c1.accurascience.com/miRecords/), miRGate (http://mirgate.bioinfo.cnio.es/miRGate/) and miRGator (http://mirgator.kobic.re.kr/). Finally, two miRNAs as targeting for APRIL gene were selected based on higher scores and better binding and the sequences of these miRNAs were obtained using the miRBase (www.mirbase.org) database.

*Primers design*

In this study, three types of primers including reverse transcription-specific stem-loop primers for the synthesis of cDNA from miRNAs, random hexamer primers for the synthesis of cDNA from mRNA and specific primers for evaluation of the expression of miRNAs and APRIL gene were used. These primers were designed by the GeneRunner and AlleleID7 softwares and the specificity of primers designed was determined by NCBI BLASTn tool. Also, the relative expression of predicted miRNAs and APRIL gene were normalized against miR-103 and GAPDH of expression, respectively. The list of used primers and their sequences illustrates in Table 2. 

*RNA isolation and cDNA synthesize*

Total RNA and miRNA were isolated from plasma samples according to the manufacturer’s protocol provided in the RNX-Plus kit (SinaClon, Iran) and quantified by NanoDrop (EPOCH2, USA). cDNA was synthesized using the mixture of 1 μg RNA, 1μM of specific stem-loop primers (for miRNAs) and random hexamer primers (for APRIL mRNA), M-MLV enzyme (Vivantis, Malaysia),1x RT-enzyme buffer and 400 μM dNTP. The mixture incubated at 75 ˚C for 5 min and then it was incubated at 25˚C for 15 min, 37˚C for 15 min, 42˚C for 45 min, and 10 min at 75˚C, in a thermal cycler system (Eppendorf, Germany). All procedures were performed in an RNase/DNasefree environment. The end, the synthesized cDNAs were stored at -20˚C.

*Quantitative real-time PCR (qRT-PCR)*

QRT-PCR was carried out to detect the expression levels of predicted miRNAs and APRIL gene in a Light Cycler 96 instrument (Roche, Germany). The reaction mixture consisted of 7.8 µL of SYBR Green PremixExRaq II (Yekta Tajhiz Azma, Iran), 1.5 µL cDNA, 0.3 µM of each forward and reverse primers (specific primers), and 5.1 µL RNase-free water with a final volume of 15 µL. The device’s temperature and time program was set in three steps, including 95ºC for 3 min, 40 cycles of 95 ºC for 10 seconds, 54 ºC for 15 seconds and 72ºC for 20 seconds for miRNAs and 95ºC for 3 min, 40 cycles of 95ºC for 10 seconds, 52ºC for 15 seconds and 72ºC for 20 seconds for APRIL and GAPDH genes. Melting curve analysis was performed after amplifications from to 60°C to 96°C with a ramp rate of 0.2°C/second and continuous fluorescence acquisition. The qRT-PCR data was measured using the comparative Cq method and the analyzed by the relative expression software tool (REST) (Pfaffl et al., 2002). All reactions were done in triplicate.

*Statistical analysis*

RT-qPCR results were analyzed with the REST software (2009). These data were presented as the mean ± standard error (SE). Receiver operating characteristic (ROC) curve analysis was carried out to investigate the diagnostic value of the miRNAs level. Additionally, the highest Youdan index was calculated. Statistical analysis was conducted using statistical package for social sciences (SPSS) (Ver 16; SSPS Inc., 184 Chicago). P-values of <0.05 were considered to indicate statistical significance.

## Results

*MiR-145-5p and miR-185-5p as miRNAs targeting APRIL gene*

**Figure 1:**
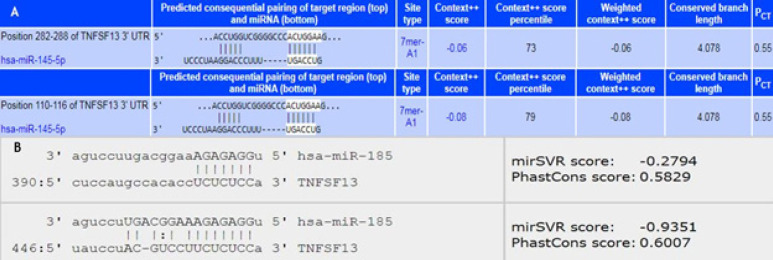
Schematic Representation of the 3`-UTRs Region of April mRNA that is Targeted by (A) miR-145-5p (in two Regions) and (B) miR-185-5p Binding Seed Region (in Two Regions)

The results obtained from different bioinformatics databases including miRanda, TargetScan, miRwalk, DIANA, miRTarBase, miRDB, miRdSNP, miRecords, miRGate and miRGator demonstrated that four miRNAs were generally capable of binding to APRIL mRNA as shown in Table 3. Although different mathematical algorithms and scoring method applied in these websites, we made a score table base on highest repeat and best complementarities with the target gene. Therefore, only miR-145-5p and miR-185-5p had the highest repeat, score and best complementarities with the target gene and these two miRNAs were chosen for the subsequent experimental study. The probability of targeting 3`-UTR part of the APRIL transcript by miR-145-5p and miR-185-5p seed reigns was shown in Figure 1.

*Expression changes of miR-145-5p, miR-185-5p and APRIL gene in plasma of patient with B-CLL*

**Figure 2:**
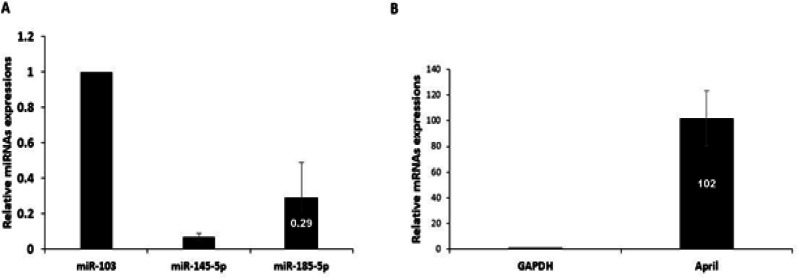
(A) Comparison of differential expression levels of miR-145-5p and miR-185-5p between patients with B-CLL and healthy individuals. (B) Relative expression of APRIL gene in B-CLL patients in comparison to healthy control group. Error bars indicate the standard error of the mean

As illustrated in Figure 2, the expression levels of miR-145-5p and miR-185-5p were significantly lower in patients with B-CLL compared to the control group. Moreover, the expression levels of APRIL gene were significantly higher in patients with B-CLL compared to control group (P= 0.001). 

*Capacity of miR-145-5p and miR-185-5p to function as new and potential biomarkers for B-CLL detection*

**Figure 3:**
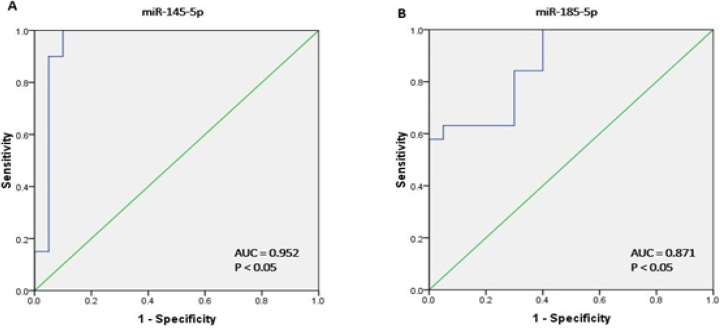
ROC Analysis was Plotted to Present the Sensitivity and Specificity of (A) the miR-145-5p expression level and (B) the miR-185-5p using area under the ROC curve (AUC) analysis

The sensitivity and specificity of role miR-145-5p and miR-185-5p as B-CLL diagnostic biomarkers was measured by ROC curves. ROC analysis revealed that at the cutoff level of 0.034, miR-145-5p had a sensitivity of 90% a specifity of 95% whit an area under the curve (AUC) of 0.95 (p< 0.05) in compression of between B-CLL patients and healthy individuals. For miR-185-5p, at the cutoff level of 0.715, the sensitivity was defined 63% and the specificity was 95%, The AUC of the ROC for miR-185-5p was 0. 87. The highest Youden indices were 0. 85 for miR-145-5p and 0.58 for miR-185-5p. Differences in the expression of miR-145-5p and miR-185-5p in patients with B-CLL and healthy individuals was shown in Figure 3. In addition, Statistical results of the comparison between the patients vs. the healthy individuals for the examined miRNAs was presented in Table 4.

**Table 1:** Baseline Characteristics of the Patients and Control Group

Parameter	Patients Number (%)	Healthy controls Number (%)
Gender		
Female	20 (50)	20 (50)
Male	20 (50)	20 (50)
Age		
Median	68	70.5
Range	60-76	62-79
peripheral blood (PB)*		
WBC count (*10^9^/L)	54.2	5.2
Hemoglobin (gr/L)	10.3	14.7
Platelet (*10^9^/L)	130	281

*, Peripheral blood

**Table 2:** Primers Used for Reverse-Transcription and RT-qPCR Assay of the Target miRNAs and APRIL

Target	Primer sequences (5`-3`)
miR-145-5p	S*: 5`- GTCGTATCGAGAGCAGGGTCCGAGGTATTCGCACTCGATACGACAGGGATT -3`
	F**: 5`- GTTTTCCCAGGAATCCCT -3`
miR-185-5p	S: 5`- GTCGTATCGAGAGCAGGGTCCGAGGTATTCGCACTCGATACGACTCAGGAA -3`
	F: 5`- GAGAGAAAGGCAGTTCCTGA -3`
miR-103	S: 5`-GTCGTATCGAGAGCAGGGTCCGAGGTATTCGCACTCGATACGACCAAGGCA-3`
	F: 5`-GCTTCTTTACAGTGCTGCC-3`
Common Reverse	5`-AGAGCAGGGTCCGAGGT-3`
APRIL	F: 5`- AACAGAAGAAGCAGCACTC -3`
	R: 5`- GCAGATAAACTCCAGCATCC -3`
GAPDH	F:5`-GGAGTCCACTGGCGTCTTCAC-3`
	R:5`-GAGGCATTGCTGATGATCTTGAGG-3`

*, Stem-loop gene-specific reverse-transcriptase primers; **, Specific forward primers for RT-qPCR amplification

**Table 3:** The Result of miRNA Prediction from Different Bioinformatics Databases for APRIL Gene

miRNA	miRTar Base	miR DB	miR Walk	Target Scan	DIA NA	miR anda	miR dSNP	miR ecords	miR Gate	miR Gator	SUM
has-miR-145-5p	1*	0	1	1	0	0	1	1	0	1	6
has-miR-185-5p	0	1	0	0	1	1	0	1	1	1	6
has-miR-6132	0**	1	0	0	1	0	0	0	1	0	3
has-miR-4644	0	1	0	0	1	0	0	0	1	0	3

*, Targeting of APRIL is confirmed by the software; **, Targeting of APRIL is not confirmed by the software.

**Table 4:** Statistical Results of the Comparison between the Patients vs. the Healthy Individuals for the Examined miRNAs

miRNA	AUC*	95% CI**	cutoff level	Y-index***	Std. Error***	sensitivity	specifity
miR-145-5p	0.95	0.870-1.035	0.034	0.85	0.042	90%	95%
miR-185-5p	0.87	0.763-0.979	0.715	0.58	0.055	63%	95%

* AUC: an area under the ROC curve; **, CI 95%: 95% confidential interval; ***, The Y-index was used to judge the most appropriate critical point. It was calculated as follow (1-[sensitivity+ specifity]); ****, Std. Error: under the non-parametric assumption.

**Figure 4:**
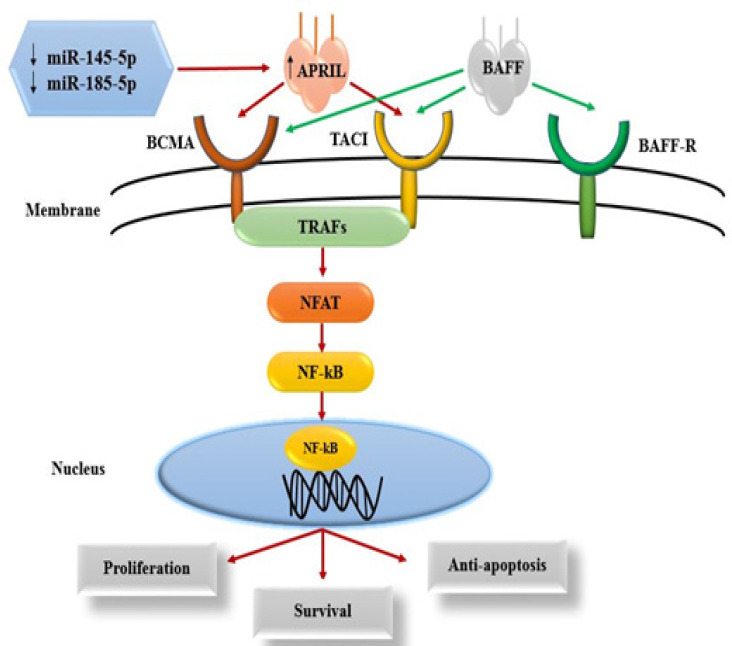
Schematic Representation of the NF-kB Signaling Pathway Activated by APRIL Ligand in the CLL Cells

## Discussion

CLL is the most prevalent leukemia in adults which remains a public health problem in the world (Fabbri and Dalla-Favera, 2016). This cancer has poor prognosis and diagnosis at different stages (Mertens and Stilgenbauer, 2014). In the past, it was difficult to diagnose B-CLL patients, but today it is partially detectable using symptoms and analysis of prognostic laboratory biomarkers (Ferrarini et al., 2012). Therefore, the identification of new biomarkers with high sensitivity and specificity is crucial for early diagnosis of B-CLL. Several reports have shown that changes in miRNAs expression are related to the development and progression of different cancers in human (Peng and Croce, 2016). Additionally, miRNAs are stable in serum and plasma, which might be due to their protection by exosomes, microvesicles and Argonaute 2 (Pan et al., 2018). Therefore, these molecules as non-invasive biomarkers can be useful for detection of different cancers including B-CLL (Fulci et al., 2007; Zanette et al., 2007). 

On the other hand, the APRIL is known as an oncogene that is rarely expressed in normal tissues but its high levels are detectable in tumor cells in vivo and in vitro and cancers such as thyroid and colon (Hahne et al., 1998b). Additionally, APRIL is a soluble molecule secreted from the Golgi apparatus (Lopez-Fraga et al., 2001). Some studies have demonstrated that APRIL, as an oncogene, upregulated in non-canonical NF-kB signaling pathway and that dysregulation of this signaling pathway contributes to development and evolution of B-CLL (Van Attekum et al., 2016). Therefore, considering the role of APRIL in the pathogenesis of B-CLL, this molecule seems to be a suitable choice for miRNAs targeting studies. 

In the present study, we predicted miRNAs targeting APRIL gene using bioinformatics software, and determined the expression levels of these miRNAs and the APRIL gene using RT-qPCR in the plasma samples of B-CLL patients and healthy individuals. ROC curve analyses were also performed to investigate the possibility of using plasma levels of predicted miRNAs as new and potential biomarkers for detecting B-CLL. 

The bioinformatics predictions presented here indicated that four miRNAs including miR-145-5p, miR-185-5p, miR-6132 and miR-4644 could target the transcript of the APRIL gene. However, miR-145-5p and miR-185-5p had higher scores and were chosen for experimental study. 

The results of RT-qPCR in the current study showed that the plasma levels of these two miRNAs were significantly lower in the B-CLL patients than in the healthy individuals. The expression of miR-145-5p and miR-185-5p were downregulated by -14.28 fold (P= 0.001) and -3.44 fold (P= 0.001), respectively. On the other hand, the plasma levels of APRIL were upregulated by 102 fold (P= 0.001) in the B-CLL patients than in the healthy individuals. The decreased expression of miR-185 has been reported in the several solid tumors (Volinia et al., 2006). Zanette et al., showed that five miRNAs including miR-135b, miR199s, miR-142-5p, miR-181c and miR-185 had the lowest expression levels in CLL (Zanette et al., 2007). The role of miR-185 has been investigated in various cancers including lung cancer, osteosarcoma and breast cancer by targeting SOX9, HK2, DNMT1 and E2F6, respectively (Tang et al., 2014; Lei et al., 2018; Liu et al., 2019). On the other hand, miR-145 was reported as a tumor suppressor miRNA because its expression is reduced in various tumor, including colon, breast, ovarian, CLL and burkitt lymphoma (Akao et al., 2007; Sachdeva et al., 2009). These finding suggest that miR-145-5p and miR-185-5p act as a tumor suppressor in the B-CLL by direct-targeting of APRIL mRNA. Therefore, B-CLL progression may be prevented by the expression of miR-145-5p and miR-185-5p in cancer cells through decreases in the expression of APRIL gene. The accuracy of miR-145-5p and miR-185-5p levels to discriminate between patients with B-CLL and healthy individuals using ROC curve analyses revealed that these two miRNAs are specific and sensitive for the detection of B-CLL but miR-145-5p presented the greatest AUC, sensitivity, and specificity as 90%, 95%, and 0.952, respectively, at a cutoff of 0.034. Therefore, differences in the expression of miR-145-5p could distinguish healthy individuals from patients with B-CLL. Akao et al., also confirmed that miR-145-5p can be used as biomarker to differentiate normal cells from malignant B cells (Akao et al., 2007). 

The results of the present study demonstrated a negative correlation between downregulation of miR-145-5p and miR-185-5p with upregulation of APRIL in B-CLL patients. This correlation may be due to the targeting of the APRIL mRNA (as an oncogene) by miR-145-5p and miR-185-5p (as tumor suppressor miRNAs).

In conclusion, these miRNAs may be serve as new and non-invasive biomarkers in the diagnosis of B-CLL. The mechanism of action of these two miRNAs is shown in Figure 4. Our study has some limitations such as Luciferase assay, to discover the exact functional relationship between identified miRNAs and the APRIL gene.

## References

[B1] Akao Y, Nakagawa Y, Kitade Y (2007). Downregulation of microRNAs-143 and -145 in B-cell malignancies. Cancer Sci.

[B2] Ardekani AM, Naeini MM (2010). The role of microRNAs in human diseases. Avicenna J Med Biotechnol.

[B3] Eichhorst B, Robak T, Montserrat E (2015). Chronic lymphocytic leukaemia: ESMO Clinical Practice Guidelines for diagnosis, treatment and follow-up. Ann Oncol.

[B4] Fabbri G, Dalla-Favera R (2016). The molecular pathogenesis of chronic lymphocytic leukaemia. Nat Rev Cancer.

[B5] Ferrarini M, Cutrona G, Neri A (2012). Prognostic factors in CLL. Leuke Suppl.

[B6] Ferrer G, Montserrat E (2018). Critical molecular pathways in CLL therapy. Mol Med.

[B7] Fulci V, Chiaretti S, Goldoni M (2007). Quantitative technologies establish a novel microRNA profile of chronic lymphocytic leukemia. Blood.

[B8] Hahne M, Kataoka T, Schröter M (1998a). APRIL, a new ligand of the tumor necrosis factor family, stimulates tumor cell growth. J Exp Med.

[B9] Hahne M, Kataoka T, Schröter M (1998b). APRIL, a new ligand of the tumor necrosis factor family, stimulates tumor cell growth. J Exp Med.

[B10] Haiat S, Billard C, Quiney C (2006). Role of BAFF and APRIL in human B-cell chronic lymphocytic leukaemia. Immunology.

[B11] Hallek M, Cheson BD, Catovsky D (2008). Guidelines for the diagnosis and treatment of chronic lymphocytic leukemia: a report from the International Workshop on Chronic Lymphocytic Leukemia updating the National Cancer Institute–Working Group 1996 guidelines. Blood.

[B12] Lei Z, Shi H, Li W (2018). miR185 inhibits nonsmall cell lung cancer cell proliferation and invasion through targeting of SOX9 and regulation of Wnt signaling. Mol Med Reports.

[B13] Liu C, Cai L, Li H (2019). miR185 regulates the growth of osteosarcoma cells via targeting Hexokinase 2. Mol Med Reports.

[B14] Lopez-Guerra M, Colomer D (2010). NF-κB as a therapeutic target in chronic lymphocytic leukemia. Exp Opinion Ther Targets.

[B15] Lopez-Fraga M, Fernandez R, Albar J (2001). Biologically active APRIL is secreted following intracellular processing in the Golgi apparatus by furin convertase. EMBO Rep.

[B16] Lu J, Getz G, Miska EA (2005). MicroRNA expression profiles classify human cancers. Nature.

[B17] Mackay F, Ambrose C (2003). The TNF family members BAFF and APRIL: the growing complexity. Cytokine Growth Factor Rev.

[B18] Mirzaei H, Fathullahzadeh S, Khanmohammadi R (2018a). State of the art in microRNA as diagnostic and therapeutic biomarkers in chronic lymphocytic leukemia. J Cell Physiol.

[B19] Mirzaei H, Momeni F, Saadatpour L (2018b). MicroRNA: relevance to stroke diagnosis, prognosis, and therapy. J Cell Physiol.

[B20] Mitchell PS, Parkin RK, Kroh EM (2008). Circulating microRNAs as stable blood-based markers for cancer detection. Proc Nat Academy Sci U S A.

[B21] Moussay E, Wang K, Cho J-H (2011). MicroRNA as biomarkers and regulators in B-cell chronic lymphocytic leukemia. Proc Natl Acad Sci U S A.

[B22] Pan JH, Zhou H, Zhao XX (2018). Role of exosomes and exosomal microRNAs in hepatocellular carcinoma: Potential in diagnosis and antitumour treatments. Int J Mol Med.

[B23] Peng Y, Croce CM (2016). The role of MicroRNAs in human cancer. Signal Transduct Target Ther.

[B24] Pfaffl MW, Horgan GW, Dempfle L (2002). Relative expression software tool (REST©) for group-wise comparison and statistical analysis of relative expression results in real-time PCR. Nucleic Acids Res.

[B25] Rodrigues CA, Gonçalves MV, Ikoma MRV (2016). Diagnosis and treatment of chronic lymphocytic leukemia: recommendations from the Brazilian Group of Chronic Lymphocytic Leukemia. Rev Bras Hematol Hemoter.

[B26] Rushworth SA, Murray MY, Barrera LN (2012). Understanding the role of miRNA in regulating NF-κB in blood cancer. Am J Cancer Res.

[B27] Sachdeva M, Zhu S, Wu F (2009). p53 represses c-Myc through induction of the tumor suppressor miR-145. Proc Natl Acad Sci U S A.

[B28] Simonian M, Mosallayi M, Mirzaei H (2018). Circulating miR-21 as novel biomarker in gastric cancer: diagnostic and prognostic biomarker. J Cancer Res Ther.

[B29] Strati P, Shanafelt TD (2015). Monoclonal B-cell lymphocytosis and early-stage chronic lymphocytic leukemia: diagnosis, natural history, and risk stratification. Blood.

[B30] Tang H, Liu P, Yang L (2014). miR-185 suppresses tumor proliferation by directly targeting E2F6 and DNMT1 and indirectly upregulating BRCA1 in triple-negative breast cancer. Mol Cancer Ther.

[B31] Tecchio C, Nichele I, Mosna F (2011). A proliferation-inducing ligand (APRIL) serum levels predict time to first treatment in patients affected by B-cell chronic lymphocytic leukemia. Eur J Haematol.

[B32] Volinia S, Calin GA, Liu C-G (2006). A microRNA expression signature of human solid tumors defines cancer gene targets. Proc Natl Acad Sci U S A.

[B33] Wang L, Zhang S, Xu Z (2017). The diagnostic value of microRNA-4787-5p and microRNA-4306 in patients with acute aortic dissection. Am J Transl Res.

[B34] Zanette D, Rivadavia F, Molfetta GAd (2007). miRNA expression profiles in chronic lymphocytic and acute lymphocytic leukemia. Braz J Med Biol Res.

